# Cortical Activation Patterns of Different Masking Noises and Correlation With Their Masking Efficacy, Determined by Functional Near-Infrared Spectroscopy

**DOI:** 10.3389/fnhum.2020.00149

**Published:** 2020-04-28

**Authors:** Qiyang Sun, Xianren Wang, Bixue Huang, JinCangjian Sun, Jiahui Li, Huiwen Zhuang, Guanxia Xiong

**Affiliations:** ^1^Department of Otorhinolaryngology, The First Affiliated Hospital, Sun Yat-sen University, Guangzhou, China; ^2^Institute of Otorhinolaryngology, Sun Yat-sen University, Guangzhou, China

**Keywords:** NIRS, tinnitus, acoustic therapy, masking noise, auditory cortex

## Abstract

Acoustic therapy in tinnitus treatment is poorly characterized, and efficacy assessment depends on subjective descriptions. Narrow-band noise, notched sound, and white noise have positive therapeutic effects on monotonous tinnitus. Considering the tonotopic characteristics of the auditory system and the spectral characteristics of these three masking sounds, the activation pattern of the auditory cortex and the mechanism of inhibiting tinnitus may be different. This study aimed to compare the activation patterns of three spectrally different masking noises and study the correlation between the masking effects and variational amplitude of oxygenated hemoglobin (HbO) in the corresponding cortical regions. We also assessed near-infrared spectroscopy brain function imaging (NIRS) as an objective assessment tool in acoustic therapy. Patients with persistent non-pulsatile tinnitus and control volunteers without tinnitus were enrolled in this study. The subjects were seated in a sound-proof room, with two optode arrays covering the bilateral temporal lobe. Auditory stimuli were presented; stimulation sequences followed the block design: different noises appeared randomly and repeated in five cycles. Tinnitus match and residual inhibition were performed in the tinnitus group. The data analyses were conducted using the NIRS_SPM toolbox. The group analysis results showed that the narrow-band noise caused a marginally significant decrease in HbO signal in the Brodmann 21 region (BA21), while white noise caused a significant increase in HbO signal in BA21. Notched sound did not cause significant changes in the HbO signal in the temporal cortex. And none of the three masking noises caused significant changes in the HbR signal in the temporal cortex. The depth of residual inhibition induced by the narrow-band noise and white noise significantly correlated with ΔHbO in the region of interest (ROI). However, neither the depth nor duration of the residual inhibition induced by notched sound correlated with the ΔHbO. Thus, NIRS showed three cortical activation patterns induced by three different masking noises, and correlations between residual inhibition effects and change of HbO amplitude were found. NIRS could therefore be applied in objective assessment of acoustic therapy.

## Introduction

Tinnitus is defined as phantom sound perception in the absence of an external sound source and is experienced by 10–15% of the general population (Sereda et al., 2018). Tinnitus may cause anxiety, depression, sleep disorders, and deterioration of social function. The proportion of individuals who typically seek medical help for tinnitus is 1% to 2%. The mechanism of tinnitus remains unclear and likely involves a complex network comprising non-auditory centers including the posterior parietal, frontal, somatosensory, and limbic regions. Tinnitus is primarily a physio-pathological change in the auditory system; compensatory changes in the central auditory system triggered by peripheral hearing loss may be one of the key factors that underlie tinnitus (Noreña and Eggermont, 2003, 2005; Vanneste et al., 2010; Pape et al., 2014; Noreña, 2015).

To understand the mechanism of tinnitus and optimize its treatment, three key features of the normal functioning auditory system have to be considered. First, the spontaneous firing of neurons that occurs throughout the peripheral and central auditory nervous systems; even in the absence of sound in the environment, there is some level of nerve firing or spontaneous firing within the auditory nervous system (San Juan et al., 2017). Second, tonotopicity is maintained from the level of the basilar membrane of the cochlea to the central auditory structures and auditory cortex. Changes in tonotopicity with auditory system damage might be associated with tinnitus (Eggermont and Roberts, 2004). Third, neural synchrony, or the balance of excitatory and inhibitory neural firing within the central auditory system, must be maintained to preserve the neural coding of auditory input (Noreña and Farley, 2013). Excitation and inhibition are important neural functions within the auditory pathway (Pickles, 2008). When transduction occurs within the inner ear, all fibers of the auditory nerve are excited, with no inhibition. Once the fibers reach the central auditory nervous system, however, different groups of neurons respond differently to stimulation. Some groups of neurons are primarily excitatory whereas others are primarily inhibitory. Current theories of the origin of tinnitus-related to abnormalities in spontaneous firing rate, tonotopicity, and/or neural synchrony.

There are no effective drugs or surgical treatments for tinnitus; acoustic therapy has been broadly accepted as a safe and convenient choice. Besides the instant masking effect to the phantom sound, after a certain period of acoustic therapy, tinnitus loudness and negative emotion related to tinnitus reported by patients reduced (Roberts et al., 2015; Zenner et al., 2017; Schad et al., 2018; Brennan-Jones et al., 2019; Neff et al., 2019). These reports have led to the hypothesis that proper masking therapy can alter patients’ tinnitus state by affecting the remapping of the auditory cortex (Pantev et al., 2012). However, the nature of subjective tinnitus makes it difficult to evaluate through objective and quantitative indicators. The symptom of tinnitus can only be reported by the patient, and physicians give their sound prescriptions depending on personal experience and feedback from patients. Thus, current tinnitus treatment largely involves trial and error.

Objective changes caused by tinnitus have been studied to some extent. Noreña and Eggermont (2005) found that the resting state network of the hypothalamus and the Heschl’s gyrus was significantly enhanced in patients with tinnitus compared to healthy controls, and correlated with the reported tinnitus loudness. By applying Auditory Brainstem Response (ABR), Gu et al. (2012) found that the amplitudes of I and V waves in patients with tinnitus were significantly higher than those in healthy controls. Although these findings were based on strict matches between the tinnitus and control groups, and are not yet considered a diagnostic basis, they suggest the potential value of brain function study in the objective examination of tinnitus. We considered whether the effects of different masking noises could be distinguished and whether the therapeutic effects of such masking sounds could be evaluated by brain functional imaging.

According to a magnetoencephalography (MEG) study, patients with tinnitus receiving a one-year treatment of Tailor-Made Notched Music (TMNM), showed a reduction in tinnitus-related cortical activity that was significantly greater than that in the placebo-controlled patients (Okamoto et al., 2010). To date, brain functional imaging studies for masking noise have mainly focused on resting-state (Xu et al., 2015; San Juan et al., 2017). There have been no brain functional imaging studies to observe the activation patterns of different masking sounds. This could be because of incompatibility between existing brain functional imaging methods and auditory tasks, e.g., the profound noise of fMRI.

Compared with traditional brain function imaging technology, near-infrared spectroscopy brain function imaging equipment has the advantages of no ionizing radiation, electromagnetic compatibility, and low noise, which make it highly suited to application in auditory tasks (Sevy et al., 2010). Moreover, the good temporal and spatial resolution may prove useful in demonstrating the brain functional effect of acoustic therapy intuitively, which could, in turn, be applied as an objective indicator of acoustic therapy. This study aimed to use fNIRS to clarify whether different masking noises with contrasting spectra (e.g., narrow-band noise, notched sound, and white noise) induce distinguishable activation patterns and if the amplitude in the corresponding region of interest (ROI) correlates with clinical indicators (e.g., residual inhibition) in patients with tinnitus.

## Materials and Methods

### Participants

Healthy subjects in the control group were volunteers from the Sun Yat-sen University. The tinnitus group consisted of patients with tinnitus from the otolaryngology clinic of the First Affiliated Hospital of Sun Yat-sen University. After tinnitus matching test and pure tone hearing threshold test, the subjects were included according to the following inclusion and exclusion criteria (Table 1). Our otologist conducted clinical evaluation among the recruited subjects by reviewing medical history and out-patient documentation. The patients’ description of their tinnitus symptoms allowed us to determine whether they had tonal tinnitus or a possible psychotic symptom, which is usually accompanied by other psychotic symptoms like delusion and sensory disturbance. This study was approved by the ethics committee of the First Affiliated Hospital of Sun Yat-sen University. All subjects gave written informed consent following the Declaration of Helsinki.

**Table 1:** Inclusion and exclusion criteria for participants.

	Control group	Tinnitus group
Inclusion criteria	General population	Complaint of consistent tinnitus lasting for more than 3 months; tinnitus pitch falls between 4,000 Hz and 8,000 Hz
Exclusion criteria	Persistent tonal or pulse-like tinnitus; unstable hypertension; trauma history such as a temporal bone fracture; the history of middle-ear surgery; psychotic symptoms; neurological diseases such as stroke or epilepsy.	Unstable hypertension; a history of trauma such as a temporal bone fracture; the history of middle-ear surgery; psychotic symptoms; neurological disease such as stroke or epilepsy; pulse-like tinnitus; severe sensorineural or conductive hearing loss.

### Audiometric Evaluation and Tinnitus Test

The hearing threshold was measured for both groups at a frequency range of 250–8,000 Hz with calibrated pure tone audiometry in a soundproof audio booth. For patients with tinnitus, tinnitus matching was performed to identify the tinnitus pitch and residual inhibition caused by three different masking sounds. The patients with tinnitus underwent tinnitus matching in a soundproof room, where the Tinnifit (Bozy Medical Technology Limited, Foshan, China) was used to play pure tones at different frequencies through headphones. We asked the patients if the exogenous sound was similar to their tinnitus, and thereby determined the tinnitus pitch of each patient. The residual inhibition, a temporary suppression of tinnitus after the termination of the external masking noise, was then determined. We played three masking sounds through the speaker directly in front of the patient at 70 dB SPL for 20 s. The patients were then asked whether they experienced a temporary suppression of tinnitus, and how long this suppression lasted. This information was used to determine residual inhibition. For example, if a patient reported that tinnitus was suppressed by 50% for 10 s, then the depth and duration of residual inhibition was recorded as “50%*10 s.” Some patients could have a tinnitus rebound when the masking sound ended (e.g., 50% louder and lasted for 10 s; in this case, the residual inhibition was recorded as −50%*10 s).

### Auditory Stimuli and Presenting Procedure

**Figure 1:**
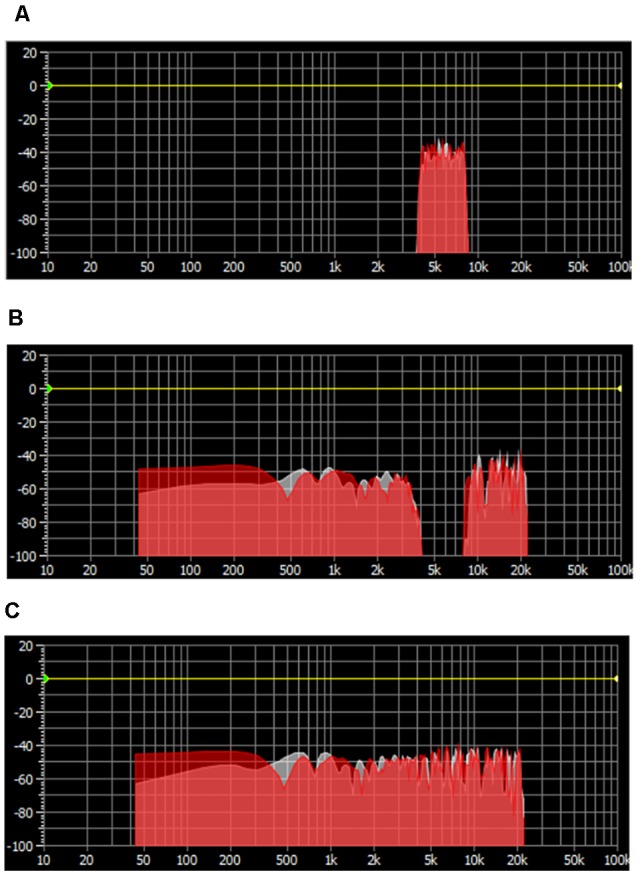
Spectrum distribution of three masking noises. **(A)** Narrow-band noise with energy clustering around 6,000 Hz. **(B)** Notched sound with an “Energy Gap” centered at 6,000 Hz. **(C)** White noise with an equally distributed spectrum energy.

Three masking sounds, which are widely used in clinical practice, were chosen as auditory stimuli. The white noise was generated as a basic tone by using Adobe Audition, and the narrow-band noise and notched sound were created by band-passing and band-stopping the 4,000 Hz–8,000 Hz spectrum, respectively (Figure 1).

**Figure 2:**
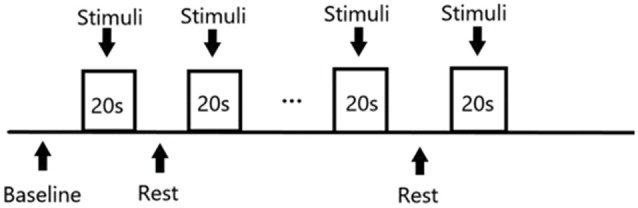
Design of stimuli presentation.

The stimulation of the study were presented through the Eprime2.0 (Psychology Software Tools, Inc., Pittsburgh, PA, USA) tool. The stimulation sequence followed a block design (Figure 2): Each of the three masking sounds ran for five blocks, where one block consisted of 20 s stimulation alternating with 20 s silence. The program therefore comprised 15 blocks and a 20 s baseline, with a total duration of 620 s. Moreover, considering the effect of decreased attention and inadvertent motion of participants, these blocks were distributed randomly to avoid uneven influence upon the results of different masking sounds.

During the auditory stimulation, the subjects were asked to listen to the masking noises passively; they were not required to make any response or remember the sequence of different noises.

### Test Environment and Optode Localization

**Figure 3:**
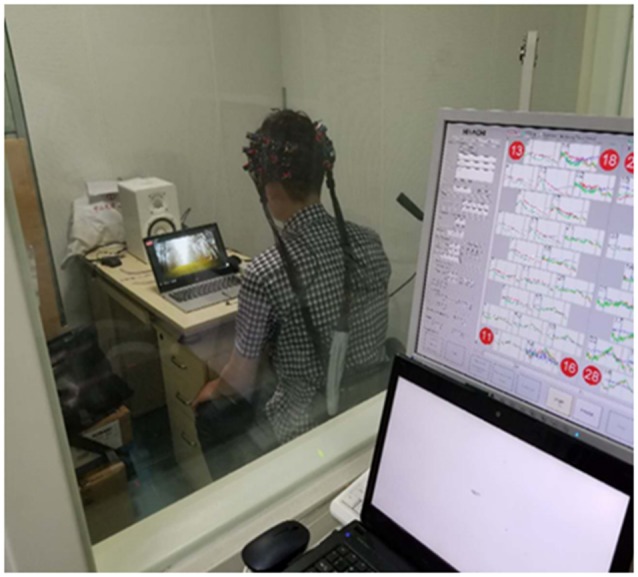
Testing facility and environment.

**Figure 4:**
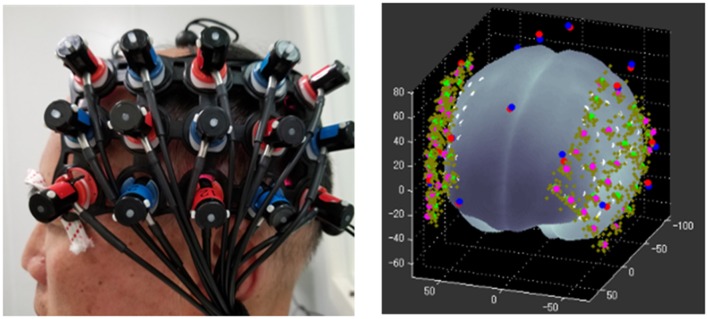
Optode array and spatial registration.

Experiments were conducted in a soundproof room (Figure 3) with background noise less than 30 dB SPL. Auditory stimuli were presented through a sound speaker placed 75 cm in the front of the subject. The sound intensity was coordinated at 70 dB SPL. All data were acquired on a Hitachi ETG4000 (Hitachi Medical Corporation, Tokyo, Japan) optical topography system with 30 optodes (16 sources and 14 detectors) arranged in two pre-defined 3*5 holder arrays, ensuring sources and detectors at fixed separations of 3 cm. These two arrays were placed over the left and right temporal regions aiming to mainly cover the bilateral auditory cortex, while the frontal and parietal lobe was involved. Infrared light was produced at two wavelengths (695 nm and 830 nm), and sampled with a frequency of 10 Hz. As shown in Figure 4, to standardize array placement, the optode arrays were placed with the central optode directly above the preauricular point on the line connecting T3/T4 and CZ using the 10-20 system (Niedermeyer and Lopes da Silva, 2005).

Considering the optical characteristics of the fNIRS device, poor contact between optodes and the skull is a major source of artifacts. To improve signal quality, we carefully removed unnecessary hair at contact points, adjusted the angle of optodes, and ran the signal check program pre-installed in the ETG 4000. We did not start the next step until all the channels passed the signal check. Another source of artifacts is motion (e.g., slight head moment) of the subject. This motion could cause changes in the blood flow, which leads to fluctuations in the measured hemodynamic response. A crude way to correct for motion artifacts is to average a certain number of experiments.

### Data Preprocessing and Statistical Analyses

The fNIRS data analysis was performed using the NIRS_SPM toolbox (Tak and Ye, 2011; Bio Imaging Signal Processing Lab. Department of Bio and Brain Engineering, KAIST, Korea). Initially, optical density data were converted into concentration changes of oxy- and deoxy-hemoglobin by using the modified Beer–Lambert law (MBLL) with (Delpy et al., 1988). We then used the time series analysis routine, comprising temporal smoothing using canonical hemodynamic response function (hrf) and wavelet-MDL detrending algorithm (Jang et al., 2009), to remove unknown global trends due to breathing, cardiac, vaso-motion, or other experimental errors. After the definition of the onset point and duration of each stimulus block, the individual analysis ran automatically. The fNIRS response time courses were fit to a general linear model (GLM) of the stimulus time-course convolved with a canonical hemodynamic response function implemented in SPM 8 software (Wellcome Trust Centre for Neuroimaging, UCL, UK, 2009), resulting in the calculation of the optimal parameter estimate (beta value) of the contribution of the stimulus to the response. Beta value estimates were calculated for each participant, channel, and stimulus separately. Beta values were then averaged across each ROI and shown as ΔHbO, providing a measure of each participant’s response to each of the three masking sounds. Group analyses were then conducted to obtain activation maps of the three different stimuli.

Statistical analysis was conducted using SPSS (version 20.0; SPSS Inc., Chicago, IL, USA). Multivariate linear regression was performed to determine the effect of age and hearing threshold on HbO response. Multivariate analysis of variance was performed to determine the main effect and interaction effect of factors such as masking noise, group, ROIs, and hemisphere. All these factors were integrated evenly, and the LSD’s method was used to adjust the *p*-values for multiple comparisons. Pearson correlations were then computed between the activation levels and the residual inhibition data. In all analyses, *P* < 0.05 was taken to indicate statistical significance.

### Definition of ROI

In this study, two ROIs were selected based on previous reports on the auditory cortex and the group analysis activation image. The ROIs were the bilateral STC and the bilateral BA21 zone. The bilateral STC is a classical audiology task interest area (including channels 7, 11, 12, 29, 33, and 34), and the BA21 area was drawn according to the activation pattern of different acoustic stimuli in this study (including channels 1, 2, 3, 23, 24, and 25).

## Results

### Clinical Data

**Figure 5:**
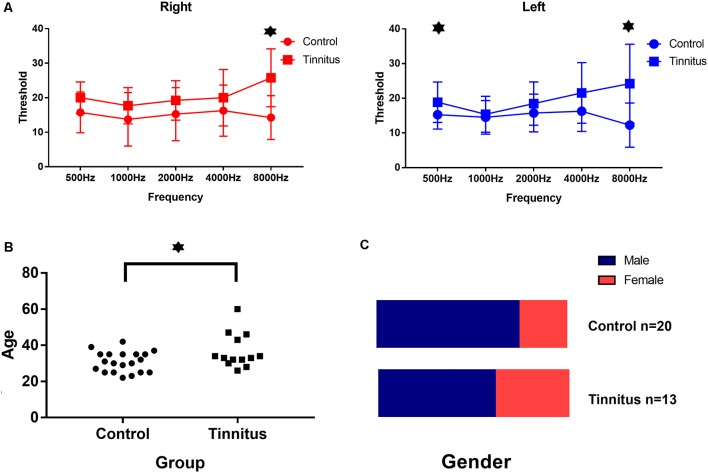
Clinical data in both group. **(A)** The average hearing threshold at 8,000 Hz for both ears and at 500 Hz for the left ear in the tinnitus group was significantly higher than that in the control group. **(B)** The average age of the tinnitus group was 37 ± 9 years and the average age of the control group was 31 ± 6 years: the difference was significant. **(C)** There was no significant difference in the ratio of gender between both two groups. ^⋆^Statistically significant/*p* < 0.05.

The relevant clinical data are presented in Tables s 2, 3, and Figure 5. There were significant differences in age and average hearing threshold at 8,000 Hz between the control and tinnitus groups. However, multivariate linear regression results showed that the effect of age (β = −0.24, *t* = −1.277, *p* = 0.212) and average hearing threshold in 8,000 Hz (Left ear: β = −0.069, *t* = −0.201, *p* = 0.842; Right ear: β = 0.229, *t* = 0.646, *p* = 0.523) on HbO response were insignificant.

**Table 2:** Age and hearing threshold in the control and tinnitus groups.

Group	Control	Tinnitus	*X*^2^	*p*
	($\bar{x}$ ± SD)	($\bar{x}$ ± SD)		
Age (year)	30.85 ± 5.68	36.77 ± 9.56	9.622	0.048
R500 Hz (dB HL)	15.75 ± 5.91	20.00 ± 4.56	5.629	0.235
R1000 Hz (dB HL)	13.75 ± 7.76	17.69 ± 5.25	3.475	0.667
R2000 Hz (dB HL)	15.25 ± 7.69	19.23 ± 5.72	3.683	0.882
R4000 Hz (dB HL)	16.25 ± 7.41	20.00 ± 8.17	4.515	0.508
R8000 Hz (dB HL)	14.25 ± 6.34	25.77 ± 8.38	13.182	0.036
L500 Hz (dB HL)	15.25 ± 4.13	18.85 ± 5.83	9.183	0.038
L1000 Hz (dB HL)	14.5 ± 4.84	15.39 ± 5.19	2.958	0.652
L2000 Hz (dB HL)	15.75 ± 5.45	18.46 ± 6.25	5.179	0.398
L4000 Hz (dB HL)	16.25 ± 5.82	21.54 ± 8.75	9.343	0.151
L8000 Hz (dB HL)	12.25 ± 6.38	24.23 ± 11.34	17.706	0.003

**Table 3:** Tinnitus matching and residual inhibition.

				Residual inhibition (Depth*Duration)		
Number	Tinnitus pitch	Sides	Duration (months)	Narrow-band noise	Notched sound	White noise
Subject 1	6,000 Hz	B	12	30%*5s	−20%*1s	30%*4s
Subject 2	6,000 Hz	B	36	20%*3s	0	50%*4s
Subject 3	6,000 Hz	B	6	20%*5s	0	20%*1s
Subject 4	6,000 Hz	B	6	60%*2s	−10%*1s	20%*5s
Subject 5	6,100 Hz	B	6	30%*3s	−10%*1s	100%*2s
Subject 6	6,000 Hz	B	12	10%*1s	0	10%*1s
Subject 7	6,000 Hz	R	3	0	0	50%*4s
Subject 8	8,000 Hz	B	10	0	−20%*2s	20%*2s
Subject 9	8,000 Hz	L	60	10%*2s	0	10%*2s
Subject 10	6,000 Hz	B	48	10%*6s	0	20%*2s
Subject 11	6,000 Hz	B	3	10%*5s	0	30%*2s
Subject 12	6,000 Hz	R	5	0	0	0
Subject 13	7,336 Hz	R	5	10%*1s	−30%*1s	100%*2s

### Multivariate Analysis of Variance

A global ANOVA with the factors masking noise, group, ROIs, and hemisphere was conducted. As shown in Table 4, only the type of masking noise showed significant main effects on the HbO signal (*df* = 2, *F* = 11.759, *P* < 0.05), and no significant interaction effects were found (not shown in the table). In this case, multiple comparisons between three different masking noises were conducted. As shown in Table 6, the HbO signal in selected ROIs induced by white noise was significantly larger than that induced by the other two masking noises, while the results between narrow-band noise and the notched sound did not show significant differences. The same procedure was also applied for HbR signal, as shown in Table 5, no significant main effect or interaction effect on HbO signal was found.

**Table 4:** Results of multivariate analysis of variance (HbO).

Source of variation	SS	df	MS	F	*P*
Mask	10.303	2	5.151	11.759	0.000
Group	0.275	1	0.275	0.628	0.429
Hemisphere	0.000	0	.	.	.
ROI	0.025	2	0.012	0.028	0.972
Error	170.413	389	0.438		
Total	185.089	396			

**Table 5:** Results of multivariate analysis of variance (HbR).

Source of Variation	SS	df	MS	F	*P*
Mask	0.066	2	0.033	1.993	0.138
Group	0.011	1	0.011	0.670	0.414
Hemisphere	0.000	0	.	.	.
ROI	0.035	2	0.018	1.060	0.347
Error	6.196	372	0.017		
Total	6.760	396			

**Table 6:** Multiple comparison between three different masking noises.

	(I) mask	(J) mask	Mean	Standard error	*P*
			difference (I-J)		
					
LSD	1	2	−0.05466971	0.081471291	0.503
		3	−0.36620861*	0.081471291	0.000
	2	1	0.05466971	0.081471291	0.503
		3	−0.31153890*	0.081471291	0.000
	3	1	−0.36620861*	0.081471291	0.000
		2	−0.31153890*	0.081471291	0.000

Mask 1, 2, and 3 indicate narrow-band noise, notched sound, and white noise, respectively. *Statistically significant/*p* < 0.05.

### Activation Patterns of the Three Different Masking Noises

**Figure 6:**
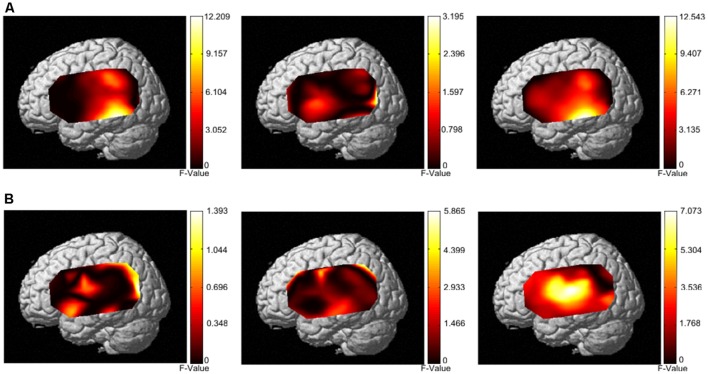
Activation patterns of the three different masking noises. **(A)** Activation patterns based on HbO data from both the control group and tinnitus group under stimulation with narrow-band noise notched sound, and white noise. **(B)** Activation patterns based on HbR data from both the control group and tinnitus group under stimulation with narrow-band noise, notched sound, and white noise.

The group analysis results from 33 individuals (Figure 6A) showed that the narrow-band noise caused a marginally significant decrease in HbO signal in the BA21 region of the bilateral temporal lobe, suggesting that the excitability of BA21 region decreased (*F* = 12.209, *P* = 0.055, Expected Euler characteristic corrected). White noise caused a significant increase in HbO signal in BA21 region, indicating that the excitability of the BA21 region increased (*F* = 12.543, *P* < 0.05, Expected Euler characteristic corrected). Notched sound did not cause significant changes in the HbO signal in the temporal cortex.

In terms of HbR results, the group analysis results from 33 individuals (Figure 6B) showed that narrow-band noise (*F* = 1.3932, *P* > 0.05, Expected Ec corrected), notched sound (*F* = 5.8659, *P* > 0.05, Expected Ec corrected) and white noise (*F* = 7.073, *P* > 0.05, Expected Ec corrected) did not cause significant changes in the HbR signal in the temporal cortex.

### Correlation Between fNIRS Data And Residual Inhibition

**Figure 7:**
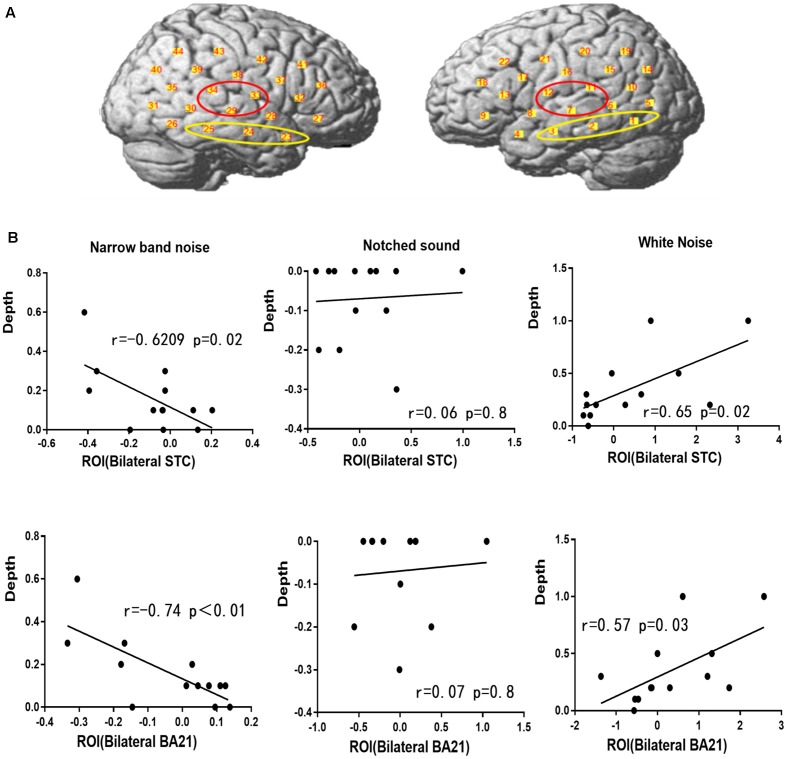
Regions of interest (ROIs) and correlation between ΔHbO and residual inhibition. **(A)** The red circle represents the bilateral STC ROI and the yellow circle represents the BA21 ROI. **(B)** Correlation between ΔHbO and residual inhibition in BA21 and STC.

Pearson correlation analysis was performed for the residual inhibition results and the amplitude of ΔHbO in each ROI (Figure 7). The depth of residual inhibition by narrow-band noise was negatively correlated with the ΔHbO in the bilateral STC (*r* = −0.6209, *p* = 0.02) and the BA21 (*r* = −0.74, *p* = 0.01). The duration was not significantly correlated with the ΔHbO amplitude in the bilateral STC (*r* = −0.2, *p* = 0.5) and BA21 (*r* = −0.35, *p* = 0.23). There was no significant correlation between the ΔHbO and depth of the residual inhibition caused by the notched sound in bilateral STC (*r* = 0.07, *p* = 0.8) and BA21 (*r* = 0.06, *p* = 0.8). The depth of the residual inhibition by white noise and the ΔHbO were positively correlated in the bilateral STC (*r* = 0.65, *p* = 0.02) and BA21 (*r* = 0.57, *p* = 0.03), and the duration of RI by the white noise showed a marginally significant correlation with the amplitude of ΔHbO in the bilateral STC (*r* = 0.52, *p* = 0.06) and BA21 areas (*r* = 0.57, *p* = 0.05).

## Discussion

Although the clinical effects of different types of masking noises have been extensively studied (Aytac et al., 2017; Rocha and Mondelli, 2017; Li et al., 2019; Tyler et al., 2020), there is no consensus on how to choose the optimal masking sound for specific therapy. Considering the clinical requirements for personalized and customized acoustic therapy, existing RCT studies on different masking sounds have limited value for clinical applications. Further studies on objective assessment for tinnitus and different masking sounds are needed to accurately predict the clinical outcome of different stimulations and conduct efficient acoustic therapy. In this study, we used a cross-sectional method to explore whether functional near-infrared spectroscopy technology could be used to detect different cortical activation characteristics of three representative masking sounds in the clinic and to determine whether the masking effect of different noises is related to the amplitude of ΔHbO in the corresponding cortical regions. Our results illustrate the potential of fNIRS technology in demonstrating the mechanism of different masking sounds and as an objective reference for clinical masking sound fitting.

There was a significant difference in mean age and the pure tone hearing threshold between the tinnitus group and the control group. Considering the control group in this study mostly comprised medical school students, the mean age and high-frequency hearing threshold failed to match. Previous studies have confirmed that different acoustic stimulation intensities (40 dB and 70 dB) induce different amplitudes of ΔHbO in the temporal cortex (Bauernfeind et al., 2016). To determine the effects of age and high-frequency hearing threshold mismatch on NIRS data, we performed multivariate linear regression analysis in this study and found no significant effect of age and hearing threshold (data not shown). Thus, although the mean age and high-frequency hearing threshold failed to match, the results of this study remain relevant.

Considering there is no significant change in HbR signal induced by three masking noise, it seems the HbO and HbR responses of three masking noise did not meet the classic definition of “activation,” which refers to an increasing HbO signal along with a decreasing HbR signal. In another fNIRS study on tinnitus patients, the HbO signal was considered as a more robust index of underlying neural activity, because of the correlations between the canonical model of the hemodynamic response function and models of HbO (vs. HbR) are consistently higher (Issa et al., 2016). Similar findings were reported from an fNIRS study on cochlear implant users, which involved only HbO data in correlation analysis with speech understanding ability (Zhou et al., 2018). Thus, the correlation analysis was based on HbO data in this study.

There has not been a detailed description and physiological explanation of the cortical activation patterns of different masking sounds. Previous functional magnetic resonance studies have shown that the area of the auditory cortex that typically responds to pure-tone stimulation of different frequencies is mainly located in the Heschl’s gyrus. The typical phoneme arrangement is characterized as low frequency at both ends and high frequency at the center of the Heschl’s gyrus (Moerel et al., 2018). However, our pre-test result showed that the high-low frequency pure tone stimulation did not yield a significant activation and the activation patterns were not completely consistent with the fMRI results (data not shown). Due to the large variance in temporal and spatial resolution between fNIRS and fMRI, and poor repeatability of cortical activation by auditory stimuli, fNIRS results cannot be directly compared to fMRI data. The inconsistency between the fNIRS and fMRI results needs further study, suggesting that the relevant activation patterns demonstrated by fNIRS should be interpreted more cautiously. Furthermore, while it is predictable that the white noise-induced activation response in the BA21, the “deactivation” induced by narrow-band noise was unexpected. BA21 is part of the temporal cortex in the human brain. The region encompasses most of the lateral temporal cortex, a region believed to play a part in auditory processing and language. Several neural imaging studies have shown functional abnormalities in BA21 among patients with chronic tinnitus (Andersson et al., 2000; Farhadi et al., 2010). White noise is believed to induced “synchrony” in the auditory cortex due to its evenly distributed energy on the spectrum, which might gradually eliminates the perception of tonal tinnitus through rebalancing the excitatory and inhibitory neural interactions within the auditory cortex (Eggermont and Roberts, 2004). In our study, white noise led to a significant increased HbO signal in BA21, which is in line with the “synchrony” theory. As for the deactivation response, a PET study (Mirz et al., 1999) showed that complex auditory stimuli like semantic material and music activated BA21 in contrast with simple auditory stimuli (e.g., pure tone), and suggested that such deactivation responses might be a manifestation of a decrease in attentional resources allocated to brain structures irrelevant to the task. However, unlike the difference between semantic material and pure tone, the major difference between narrow-band noise and white noise is the energy distribution on the spectrum. How such differences can trigger the reallocation of attentional resources in BA21 remains unclear. Thus, we were unable to completely interpret the auditory cortex activation pattern of different masking sounds in this study but showed the potential of fNIRS for the discovery of different characteristics of masking sounds in the context of brain function.

Narrow-band noise, notched sound, and white noise are the most common clinically used sounds. The residual inhibition corresponding to notched sound in this study was significantly inferior to that of narrow-band noise and white noise, and some patients reported a brief increase in tinnitus loudness after listening to the notched sound. The Tinnitus Handicap Index (THI) of the patients decreased significantly after the application of notched sound therapy, which is not in agreement with the inferiority of notched sound in residual inhibition since it is widely accepted that residual inhibition is an indicator for predicting clinical outcome in acoustic therapy. The Zwicker effect describes a short-lived tinnitus-like feeling when a person listens to a sound with a narrow-band gap in a spectrum of energy (Franosch et al., 2003). This effect may explain why the notched sound did not induce residual inhibition and even caused tinnitus to rebound. Therefore, notched sound could be an exception in terms of residual inhibition.

A major feature of subjective tinnitus is the difficulty to perform an objective and quantitative assessment, which restricts the development of treatments. As shown in our study, fNIRS data is correlated with existing behavioral indicators such as residual inhibition. The depth of residual inhibition by narrow-band noise was negatively correlated with the ΔHbO in the bilateral STC and the depth of the residual inhibition by white noise and the ΔHbO in the bilateral STC and BA21 were positively correlated. These results reveal the potential value of fNIRS as an objective indicator of masking effects. In the future, we could use fNIRS data as an objective evaluation indicator to predict the clinical outcome of acoustic therapy. However, this cross-sectional study only demonstrated the correlation between fNIRS data and the residual inhibition, which is only an indirect indicator of long-term clinical outcomes. Moreover, the mechanism of tinnitus likely involves a complex network comprising non-auditory centers, including the posterior parietal, frontal, somatosensory, and limbic regions. In this study, although the optode positions covered some of these areas, we mainly focused on the temporal region. A prospective study is needed to determine the correlation between fNIRS results and clinical outcomes.

## Data Availability Statement

The datasets generated for this study are available on request to the corresponding author.

## Ethics Statement

The studies involving human participants were reviewed and approved by The ethics committee of the First Affiliated Hospital of Sun Yat-sen University. The patients/participants provided their written informed consent to participate in this study.

## Author Contributions

QS and XW as the first authors contributed equally in the conception and design of the study and organized the database and performed the statistical analysis. QS wrote the first draft of the manuscript. BH, JL, and JS wrote sections of the manuscript. QS, XW, BH, JS, JL, HZ, and GX contributed to manuscript revision, read and approved the submitted version.

## Conflict of Interest

The authors declare that the research was conducted in the absence of any commercial or financial relationships that could be construed as a potential conflict of interest.
